# Supplementary material for: Requirements for RNA polymerase II preinitiation complex formation in vivo
Source: eLife. 2019 Jan 25;8:e43654. doi: 10.7554/eLife.43654 (PMC6366898; doi:10.7554/eLife.43654)
Supplement: Supplementary file 1. [file elife-43654-supp1.pdf]

Table S1. Yeast Strains

Strains used

Strain name	Genotype	Comment
HHY168	MATalpha, tor1-1, fpr1::NAT, RPL1:	Wildtype control strain; Haruki et al., 2008
KHW75	MATalpha, tor1-1, fpr1::NAT, RPL1:	Pol II (Rpb1) anchor-away strain
KHW76	MATalpha, tor1-1, fpr1::NAT, RPL1:	TBP (Spt15) anchor-away strain
KHW125	MATalpha, tor1-1, fpr1::NAT, RPL1:	TFIIE (Tfa1) anchor-away strain
KHW124	MATalpha, tor1-1, fpr1::NAT, RPL1:	TFIIE (Tfa2) anchor-away strain
KHW127	MATalpha, tor1-1, fpr1::NAT, RPL1:	Kin28 anchor-away strain
YJ90	MATalpha, tor1-1, fpr1::NAT, RPL1:	TFIIH (Ssl1) anchor-away strain
NP137	MATalpha, tor1-1, fpr1::NAT, RPL1:	TFIIH (Ssl2) anchor-away strain
KHW129	MATalpha, tor1-1, fpr1::NAT, RPL1:	TFIIB (Sua7) anchor-away strain
KHW130	MATalpha, tor1-1, fpr1::NAT, RPL1:	TFIIA (Toa1) anchor-away strain
KHW131	MATalpha, tor1-1, fpr1::NAT, RPL1:	TFIIA (Toa2) anchor-away strain
KHW132	MATalpha, tor1-1, fpr1::NAT, RPL1:	TFIID (Taf1) anchor-away strain
KHW134	MATalpha, tor1-1, fpr1::NAT, RPL1:	TFIIF (Tfg1) anchor-away strain
YJ127	MATalpha, tor1-1, fpr1::NAT, RPL1:	Spt16 anchor-away strain
KHW258	MATalpha, tor1-1, fpr1::NAT, RPL1:	Rgr1 anchor-away strain
